# Effect of Hygrothermal Aging and Surface Treatment on the Dynamic Mechanical Behavior of Flax Fiber Reinforced Composites

**DOI:** 10.3390/ma12152376

**Published:** 2019-07-25

**Authors:** Xiaomeng Wang, Michal Petrů

**Affiliations:** Institute for Nanomaterials, Advanced Technologies and Innovation, Technical University of Liberec, Studentska 2, 461 17 Liberec, Czech Republic

**Keywords:** flax fiber, composite, damping, hygrothermal aging

## Abstract

The recent developments of FRP (fiber reinforced polymer) are towards the growth and usage of natural FRP in the field of engineering due to both environmental and economic benefits. Flax fiber is one of the most commonly used natural fibers. One of the critical factors affecting the mechanical behavior of FFRP (flax fiber reinforced polymer) is hygrothermal aging. Some experimental works have been conducted to investigate the effect of hydrothermal aging on static behavior of FFRP. However, fewer efforts have been made to study its damping properties after hydrothermal aging. In this paper, the effect of surface treatment (including alkalization, silanization, acetylation and alkali-silanization) on dynamic mechanical behavior of FFRP under hygrothermal aging is studied. The results show that water resistance and damping properties of FFRP are improved after surface treatment. The acetylation treated FFRP exhibits excellent damping performance among all treated specimens.

## 1. Introduction

In recent years, the use of flax fiber as a potential alternative to traditional fiber such as glass fiber and carbon fiber in fiber reinforced polymer (FRP) has gained attention among researchers [1,2,3,4]. Compared to glass fiber and carbon fiber, flax fiber presents low density, low cost and easy disposal at end-of-life [5,6]. However, the porous structure of flax fiber leads to high water absorption [7,8,9]. The interaction between hydrophilic fiber and hydrophobic matrix results in weakening of the fiber/matrix interface, dimensional instability, matrix cracking and degradation of mechanical properties of the flax fiber reinforced polymer (FFRP) [10,11,12,13]. Some experimental research has been done to study the effect of moisture on the mechanical behavior of FFRP. Assarar et al. [14] and Mak et al. [15] compared the effect of environmental conditioning on the mechanical properties of FFRP and GFRP (glass fiber reinforced composite). Assarar et al. [14] reported that the saturated weight gain of FFRP is 12 times higher than that of GFRP. The weakening of fiber/matrix interface is the main damage mechanism caused by water aging of the FFRP. Mak et al. [15] found that the residual tensile strength of FFRP subjected to salt solution is higher than GFRP. After being soaked in 3.5% salt solution at 55 °C for 300 days, the residual strength of FFRP is 72% of the reference sample, while the residual strength of GFRP is 61% of the initial value. Ventura et al. [16], Scida et al. [17] and Thuault et al. [18] studied the effect of hygrothermal aging on the mechanical properties of FFRP. Ventura et al. [16] found that the water uptake of FFRP increases with flax fiber content. For example, when fiber content increases from 0% to 25%, the saturated moisture content of FFRP increases from 1.1% to 6.2%. Scida et al. [17] reported that after 38 days of hygrothermal aging with a relative humidity of 90% at 40 °C, the elastic modulus and tensile strength of FFRP decrease by 58% and 52%, respectively. Thuault et al. [18] found that the hygrothermal aging causes disorganization of the flax microfibrils network and the plasticization of the matrix, which leads to mechanical property degradation of FFRP.

Some recent research shows that the water resistance and mechanical properties of FFRP can be improved by chemical treatments such as alkalization [19,20], acetylation [21,22], and silanization [23,24]. For example, Lin et al. [19], Amiri et al. [20] and Bledzki et al. [21] reported that alkalization and acetylation help to clean the fiber surface by removing waxes, oils, and cuticle, therefore the surface morphology of the fiber surface is rougher which results in better mechanical interlocking at fiber/matrix interface. Georgiopoulos et al. [24] reported that silane works as a waterproof coating and a bridge between the fiber and matrix by introducing covalent bonds between the fiber and matrix. After silane treatment, the fiber/matrix interface strength increases, and the moisture absorption of the FFRP is reduced [25].

Damping is one of the important indicators to measure the dynamic mechanical behavior of structures [26]. However, due to a short history of FFRP in the engineering field, the effect of surface treatment on damping of FFRP under hygrothermal aging condition remains an open question. Duc et al. [27] compared the damping properties of composites with different kinds of fiber (glass fiber, carbon fiber, flax fiber) and matrix (epoxy, polypropylene, polylactide). The results show that the damping properties of flax fiber reinforced epoxy composites are better than carbon and glass fiber reinforced composites at room temperature. Adding unidirectional flax fiber to epoxy resin increases the loss factor of epoxy resin by nearly 100%. Cheour et al. [28] studied the effect of water on the damping of FFRP. The results show that loss factor of FFRP increases with soaking time. Berges et al. [29] reported that after being conditioned at 70 °C with a relative humidity of 85% for 2 weeks, a significant decrease of the storage modulus of FFRP is observed. Tayfun et al. [30] studied the effect of surface treatment on short flax fiber reinforced thermoplastic polyurethane composite. The results show that after alkali, permanganate, peroxide and silane treatment, the adhesion between short flax fibers and polyurethane is improved, and the storage modulus and glass transition temperature are increased.

Although some experimental works have been reported on the effect of surface treatment on static behavior of FFRP, research on its damping behavior is still limited. In this work, the effect of surface treatment on the damping of FFRP under hygrothermal aging conditions is studied.

## 2. Materials and Methods

**Figure 1:**
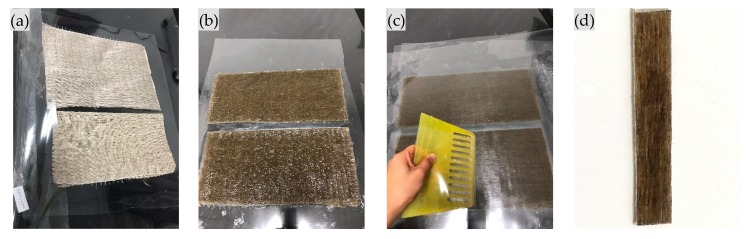
Fabrication of flax fiber reinforced polymer (FFRP) specimen: (**a**) flax fabric; (**b**) coating with epoxy; (**c**) remove bubbles; (**d**) cut into desired shape.

The unidirectional flax fabric (Figure 1a) was supplied by Nanjing Hitech Composite Co., Ltd. Epoxy (Nanjing, China) resin (E 44) and curing agent (C 650) from Nantong Xingchen Synthetic Material Co. (Nantong, China) were used as the matrix. Four kinds of chemical treatments of flax fiber were adopted: alkalization, silanization, acetylation and alkali-silanization. Alkalization is soaking the specimen in 5 wt% NaOH solution for 0.5 h at 25 °C. Silanization is soaking the specimen in 0.1 wt% silane solution (triethoxy silane) for 1 h at 25 °C. Alkali-silanization is treating with NaOH solution first, then washing with distilled water and drying in an oven, then treating with silane solution. Acetylation is soaking the specimen in glacial acetic acid for 2 h at 30 °C first, and then treating with acetic anhydride (with FeCl_3_ as catalyzer) for 1 h at 50 °C.

After surface treatment, FFRP was laminated using hand layup as shown in Figure 1. The FFRP specimen is composed of approximately 30% fiber in volume. The dimension of FFRP specimen (Figure 1d) in rectangular form is 50 mm × 10 mm × 1.35 mm. The tests were carried out according to Chinese standards GB/T 2573-2008 [31]. After curing in a laboratory environment (25 °C) for 2 weeks, specimens were dried in an oven at 60 °C for 24 h, and the initial weight of each specimen was measured. Then the specimens were soaked in distilled water at 60 °C in a test chamber. Specimens were taken out periodically and weighed to evaluate water absorption.

After 4, 9 and 16 days of hygrothermal aging, the specimens were tested to assess the damping properties. DMS (Dynamic Mechanical Spectrometer) 6100 (Hitachi High-Tech Science Corporation, Tokyo, Japan) was adopted as the test device to measure the dynamics properties of FFRP, such as storage modulus, loss modulus and loss factor. FFRP specimens were tested under three-point bending mode at a fixed frequency of 1.0 Hz. And the specimens were heated in an air atmosphere at an increased heating rate of 10 °C/min. All experimental values were obtained by an average value of three specimens.

## 3. Test Results

The water diffusion of the specimen is simplified as a one-dimensional diffusion because the length and width of the specimen are much larger than its thickness. The weight increment percentage *M_t_* of FRP in the water absorption test is defined as

(1) $$M_{t}=\frac{W_{t}-W_{0}}{W_{0}}\times 100\%$$

where *W_0_* is the initial weight, and *W_t_* is the weight after water absorption. Luo et al. [32] and Karbhari et al. [33] reported that the water absorption of FRP follows a Fickian behavior as

(2) $$\frac{M_{t}}{M_{m}}=\left\{\begin{array}{l} \frac{4}{h}\sqrt{\frac{Dt}{\pi }},\;\;\;\;\;\;\;\;\;\;\;\;\;\;\;\;\;\;\;\;\;\;\;\;\;\;\;\;\;\;\;\;\;\frac{M_{t}}{M_{m}}<0.6\\ 1-\exp\left[-7.3{\left(D\frac{t}{h^{2}}\right)}^{0.75}\right],\;\frac{M_{t}}{M_{m}}\geq 0.6 \end{array}\right.$$

where *h* is the specimen thickness, *t* is the soaking time, and *M_m_* is the maximum weight increment percentage. When $\frac{M_{t}}{M_{m}}<0.6$, the diffusivity coefficient *D* is obtained as

(3) $$D=\frac{\pi }{t}{\left(\frac{hM_{t}}{4M_{m}}\right)}^{2}.$$

**Figure 2:**
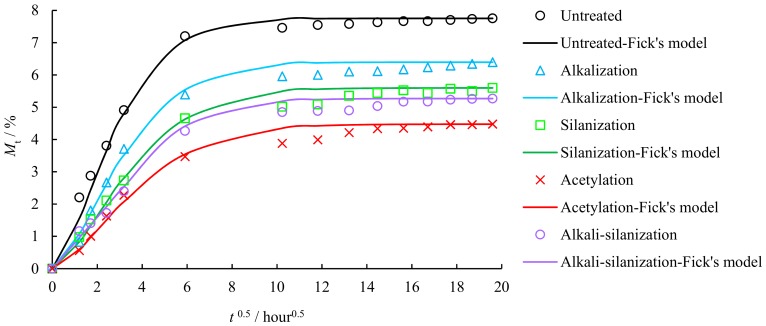
Relative water uptake of FFRP weight increment percentage of FFRP.

Figure 2 and Table 1 show the results obtained from water absorption test.

**Figure 3:**
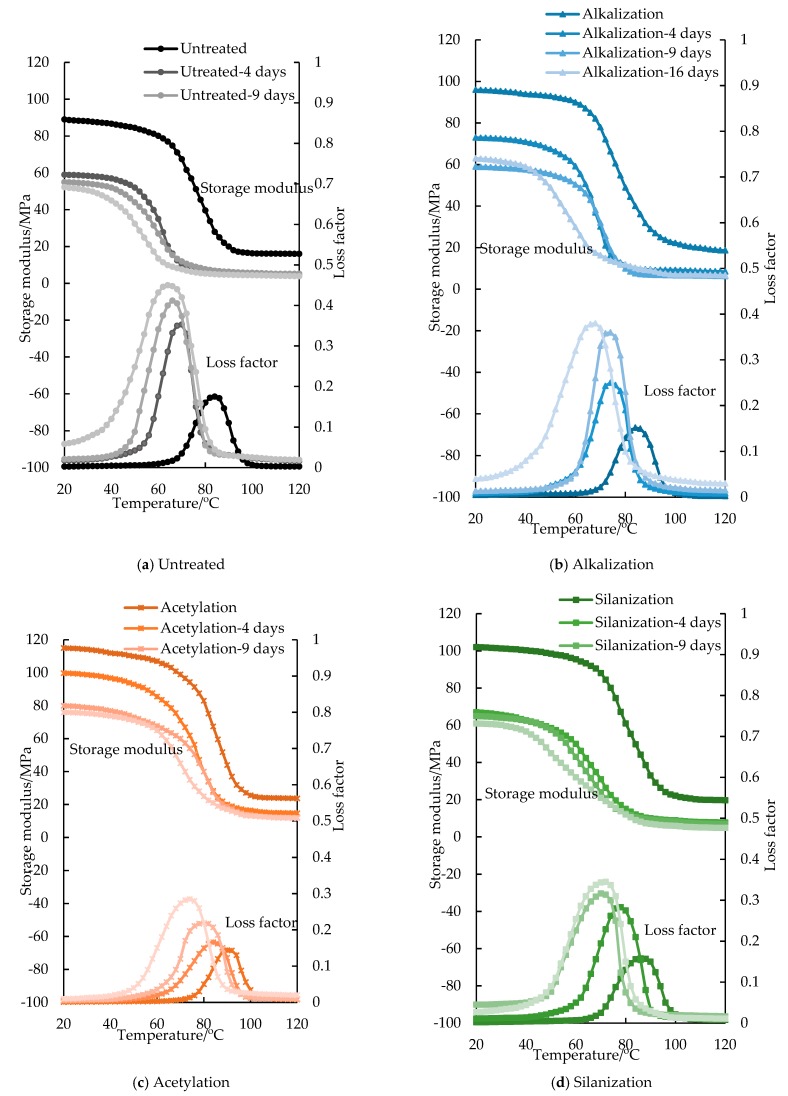

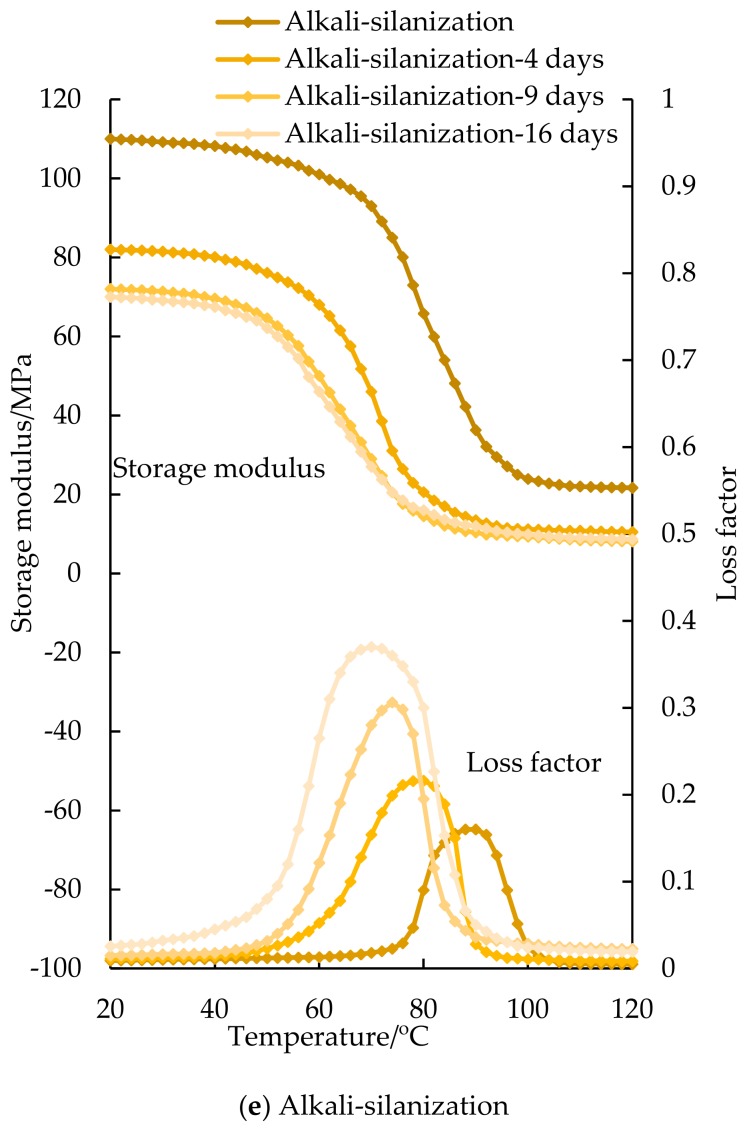
Storage modulus and loss factor of FFRP after hygrothermal aging.

**Figure 4:**
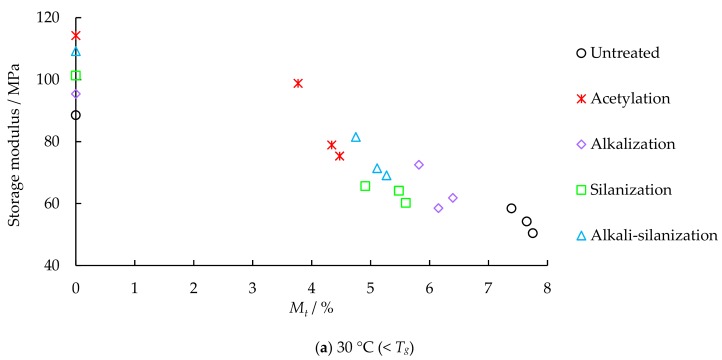

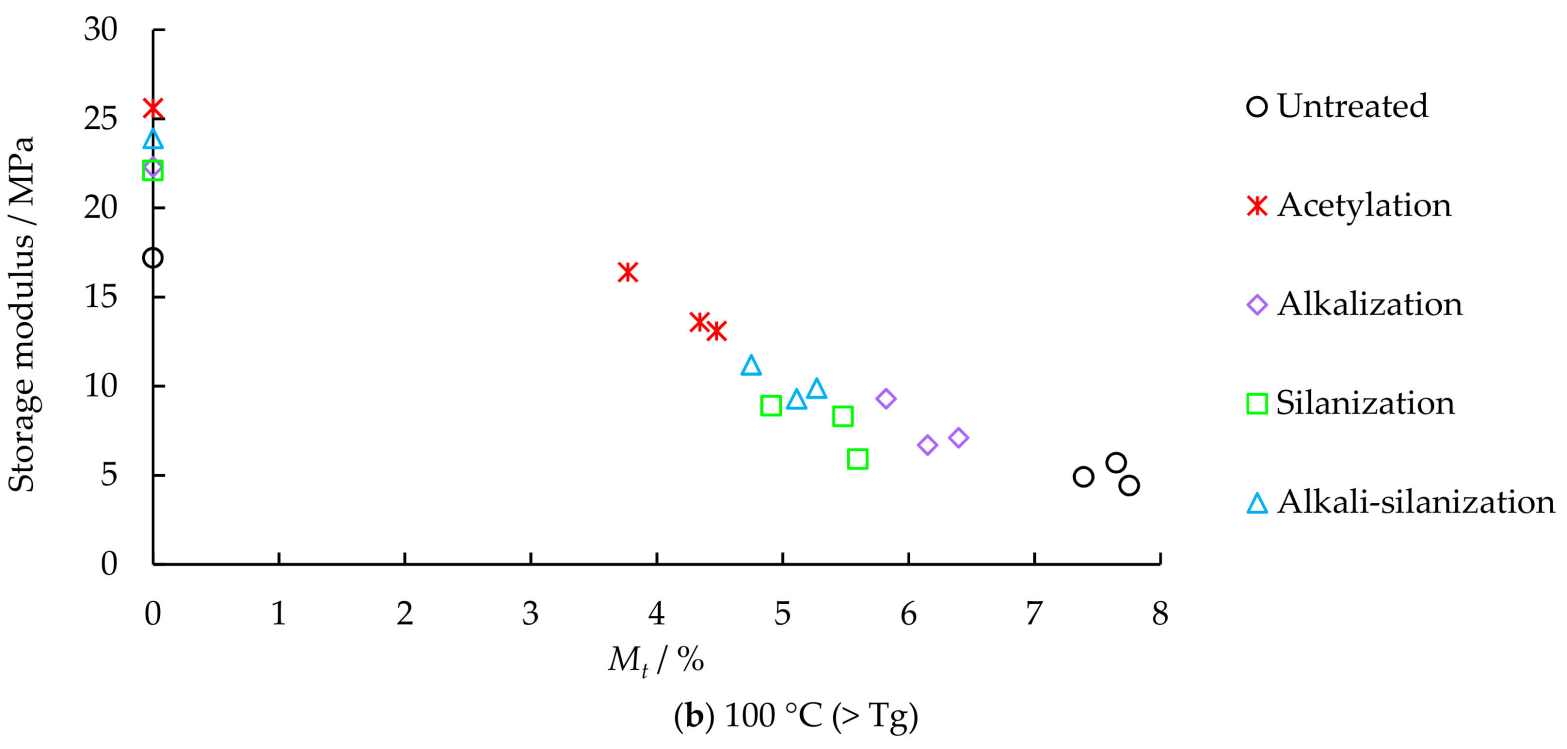
Relationship between storage modulus and moisture content.

**Figure 5:**
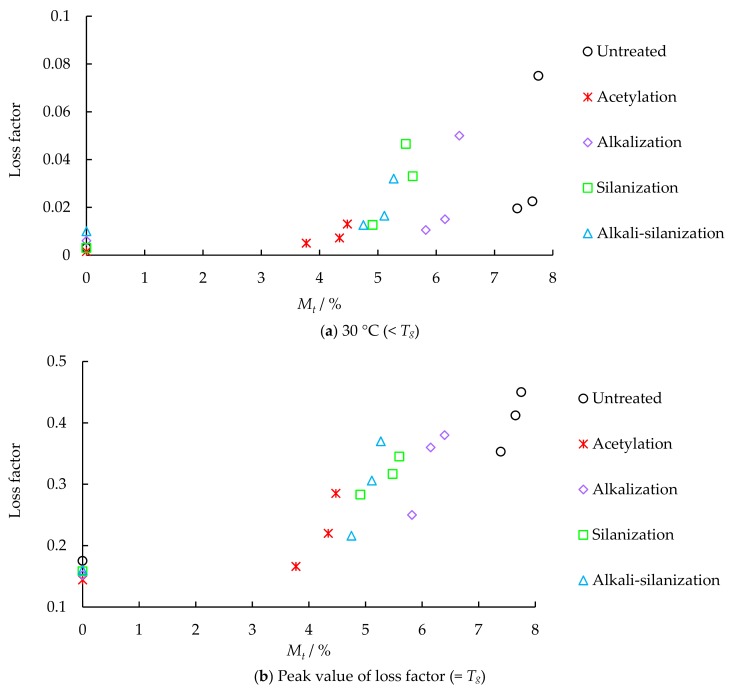
Relationship between loss factor and moisture content.

**Figure 6:**
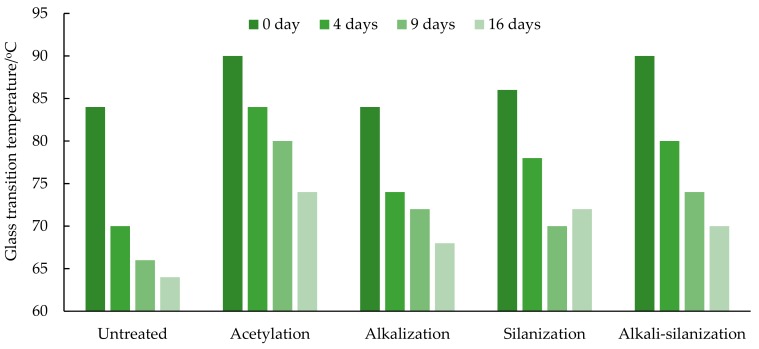
Glass transition temperature of FFRP.

The dynamic test results of FFRP under different hydrothermal aging are shown in Figure 3. The relationships between the damping properties of FFRP and moisture content are shown in Figure 4 and Figure 5. As shown in Figure 6, a significant decrease in glass transition temperature of FFRP is observed after hygrothermal aging.

## 4. Discussion

As shown in Figure 2 and Table 1, the weight increment percentage *M_t_* of FFRP increases significantly after being exposed to a hydrothermal environment, and the increasing rate slows down with the increase of aging time. The diffusion coefficient and maximum weight increment percentage of all treated specimens are lower than the reference specimen. The difference in hygroscopicity indicates that surface treatment helps slow down the rate of moisture diffusion process [34,35]. Compared with the reference specimen, the diffusion coefficient of alkalization, silanization, acetylation, and alkali-silanization treated FFRP decreases by 30.1%, 43.7%, 47.3%, 45.8%, and the maximum weight increment percentage *M_m_* decreases by 17.4%, 27.7%, 42.2%, 32.0%, respectively.

The storage modulus curve of FFRP can be divided into two regions: the glassy region (temperature below glass transition temperature *T_g_*), and the rubbery region (temperature above *T_g_*), as shown in Figure 3. In the glassy region, the components in FFRP are highly immobile, therefore the storage modulus of FFRP is high. In the rubbery region, the components of the FFRP are more mobile and lose their close packing arrangement, therefore the storage modulus decreases with temperature [36]. The storage modulus of FFRP in both the glassy region and the rubbery region decreases significantly after hydrothermal aging. This is due to the degradation of epoxy, fiber and the fiber/epoxy interface. As shown in Figure 4, moisture has significant influence on the storage modulus of FFRP. Moisture absorption increases rapidly in the initial stage, and the storage modulus decreases rapidly. With the increase of hydrothermal aging time, the moisture content tends to be saturated, and the decline rate of storage modulus also slows down. For example, after 16 days of hygrothermal aging, the storage modulus of the reference specimen at 30 °C (< *T_g_*) decreases by 51.5%, 63.3% and 75.6%, and the storage modulus of the reference specimen at 100 °C (> *T_g_*) decreases by 251.0%, 201.8%, and 290.0%, respectively. In addition, the slope of the transition zone of the storage modulus curve decreases with hydrothermal aging time.

After chemical treatment, the storage modulus of FFRP is significantly improved in both the glassy and the rubbery region. For example, after 16 days of hydrothermal aging, compared with the reference specimen, the storage modulus of the acetylation, alkalization, silanization, and alkali-silanization treated specimens at 30 °C increased by 49.4%, 22.6%, 19.4%, and 37.1%; the storage modulus at 100 °C (>*T_g_*) increases by 197.1%, 61.0%, 33.8%, and 124.5%, respectively. Firstly, chemical treatment contributes to reducing the water uptake of the FFRP. Secondly, chemical treatment helps create a stronger bond interface between fiber and matrix, which limits the movement of the molecules. Therefore, the storage modulus of FFRP is improved.

Damping represents the dissipation of energy in FFRP under cyclic loading. The loss factor is measured as the tangent of the phase angle or the ratio of loss modulus to storage modulus. As shown in Figure 3 and Figure 5, the value of the loss factor increases with hygrothermal aging time and moisture content in FFRP. For example, after 4, 9 and 16 days of hygrothermal aging, the peak value of the loss factor of the reference specimen increase by 50.4%, 57.5%, and 61.1%, respectively. It is also noticed that after hydrothermal aging, the peak point of the loss factor shifts to lower temperatures, and the curve becomes wider. Firstly, hygrothermal aging causes disorganization of flax microfibrils network and the plasticization of matrix. Secondly, water weakens the van der Waals force of the flax/matrix interface and causes the hydrolysis of chemical bonds at the interface, which leads to interfacial debonding. Previous studies [37,38,39,40] have shown that FRP with a poor fiber/matrix interface tends to dissipate more energy than FRP with good interfacial adhesion, therefore, poor interface adhesion leads to a larger loss factor.

The peak value of loss factors of treated FFRP are lower than the reference specimen. After 16 days of hygrothermal aging, the peak value of the loss factor decreases by 36.7%, 15.6%, 23.3%, and 33.9% after acetylation, alkalization, silanization and alkali-silanization. The loss factor of fiber reinforced composites mainly depends on matrix, fiber volume ratio and the fiber/matrix interface [37,41,42]. Since all specimens have the same matrix and fiber volume ratio, acetylation and alkali-silanization treated specimens exhibit the lowest peak value of loss factor, which indicates that flax fiber after these treatments show better adhesion with the epoxy. As mentioned in the introduction, acetylation and alkalization help to clean the impurities and pollutants, and promise a rougher fiber surface to provide mechanical interlocking between fiber and matrix. Therefore, flax fiber hinders the mobility of polymer chains due to the enhancement of the fiber/matrix interface and the loss factor is reduced.

As shown in Figure 6, a significant decrease in the glass transition temperature of FFRP is observed after hygrothermal aging. For example, the glass transition temperature of the reference specimen decreases by 20.0%, 27.3%, 31.3% after 4 days, 9 days and 16 days of hygrothermal aging. Moisture has an adverse effect on the molecular structure of epoxy. Water can loosen the molecular chains of epoxy, increase the molecular spacing, and enhance the activity of chemical groups, which results in the decrease of glass transition temperature [43]. Water also reduces the intermolecular hydrogen bonds between flax fiber and epoxy by creating intermolecular hydrogen bonds between the cellulose molecules and water molecules. At first, water absorbs quickly, which leads to a significant drop in the glass temperature of FFRP. With the increase of hygrothermal aging time, the moisture content of FFRP tends to be saturated and the decrease rate of the glass temperature slows down.

An increase of the glass transition temperature of FFRP is observed after surface treatment. Compared with reference specimen, the value of the glass transition temperature increases by 15.6%, 6.3%, 12.5%, 9.4% after being treated by acetylation, alkalization, silanization and alkali-silanization, respectively. It can be inferred that this is a manifestation of structural enhancement after surface treatment. Systems with more restrictions and greater enhancements tend to exhibit higher glass transition temperatures [44]. After chemical treatment, the flax fiber surface is cleaner and rougher. The change of surface morphology leads to larger contact area between the fibers and epoxy and a stronger fiber/matrix bond interface, which limits the molecular mobility of FFRP when exposed to elevated temperatures.

## 5. Conclusions

Previous research shows that the static mechanical properties and water resistance of FFRP can be improved after surface treatment. The tests carried out in this paper show that surface treatments are also critical in improving the damping properties of FFRP under hydrothermal conditions.

Test results show that the storage modulus and glass transition temperature of FFRP decrease significantly with hygrothermal aging time and moisture content. The decreasing rate slows down with the increase of aging time. In addition, an increase of the peak value of the loss factor is observed. After 16 days of hygrothermal aging, the peak value of the loss factor of the reference specimen increases by 61.1%, and the glass transition temperature decreases by 31.3%. This is mainly due to the plasticization of epoxy resin and the degradation of the fiber/matrix interface.

After surface treatment, the water uptake of FFRP is reduced. The diffusion coefficient of the FFRP after alkalization, silanization, acetylation, and alkali-silanization decreases by 30.1%, 43.7%, 47.3%, 45.8%, respectively. Compared with reference specimen, the storage modulus and glass transition temperature of the treated specimen are higher, and the peak value of the loss factor is lower. The acetylation treated FFRP exhibits excellent damping performance in all dynamic properties.

Environmental conditions are one of the most critical factors affecting the service life of FRP. Therefore, it is necessary to consider the effects of environmental degradation on the FFRP when evaluating the overall performance of the FRP structures. The test carried out in this paper may help increase the durability database and achieve a more fundamental understanding of the degradation mechanism of FFRP. The obtained relationships between moisture content and damping properties may have potential applications in life predictions of FFRP structures.

## Figures and Tables

**Table 1:** Maximum weight increment percentage *M_m_* and diffusion coefficient *D* of FFRP.

Surface Treatment	Untreated	Alkalization	Silanization	Acetylation	Alkali-Silanization
*M_m_* (%)	7.75	6.40	5.60	4.48	5.27
*D* (10^−6^ mm^2^/s)	4.19	2.93	2.36	2.21	2.27
