# Correction: Effect of Hygrothermal Aging and Surface Treatment on the Dynamic Mechanical Behavior of Flax Fiber Reinforced Composites. *Materials* 2019, *12*(15), 2376

**DOI:** 10.3390/ma12203405

**Published:** 2019-10-17

**Authors:** Xiaomeng Wang, Michal Petrů

**Affiliations:** Institute for Nanomaterials, Advanced Technologies and Innovation, Technical University of Liberec, Studentska 2, Liberec 461 17, Czech Republic; Xiaomeng.Wang@tul.cz

The Y-axis in both Figure 3 and Figure 4 of [1] were wrongly drawn when the authors output the test data to the software to form figures. Therefore, the authors wish to make the following correction to Figure 3 and Figure 4:

## Figures and Tables

**Figure 3:**
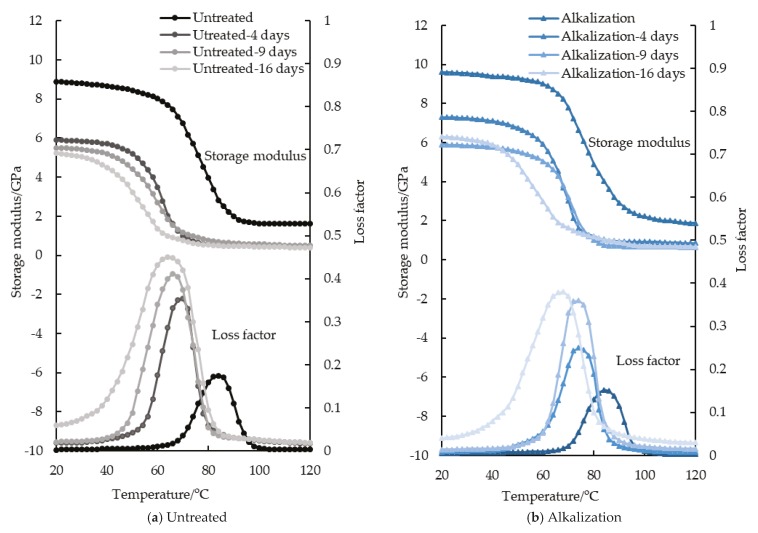

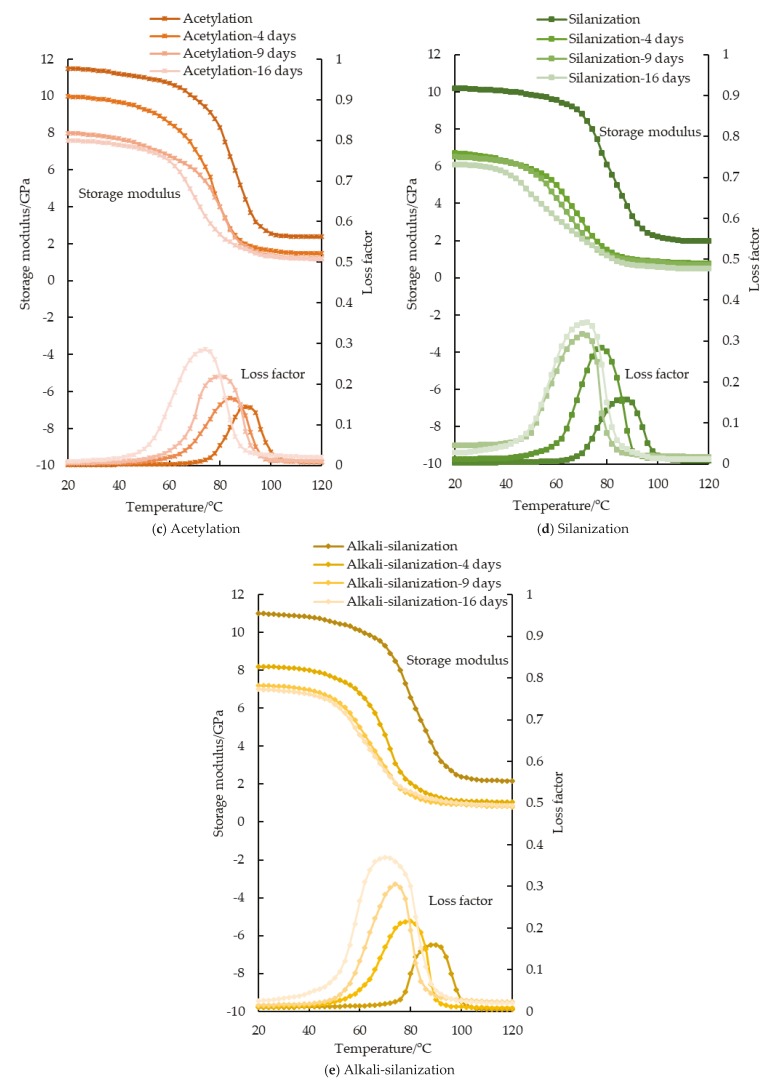
Storage modulus and loss factor of FFRP (Flax fiber Reinforced Polymer) after hygrothermal aging.

**Figure 4:**
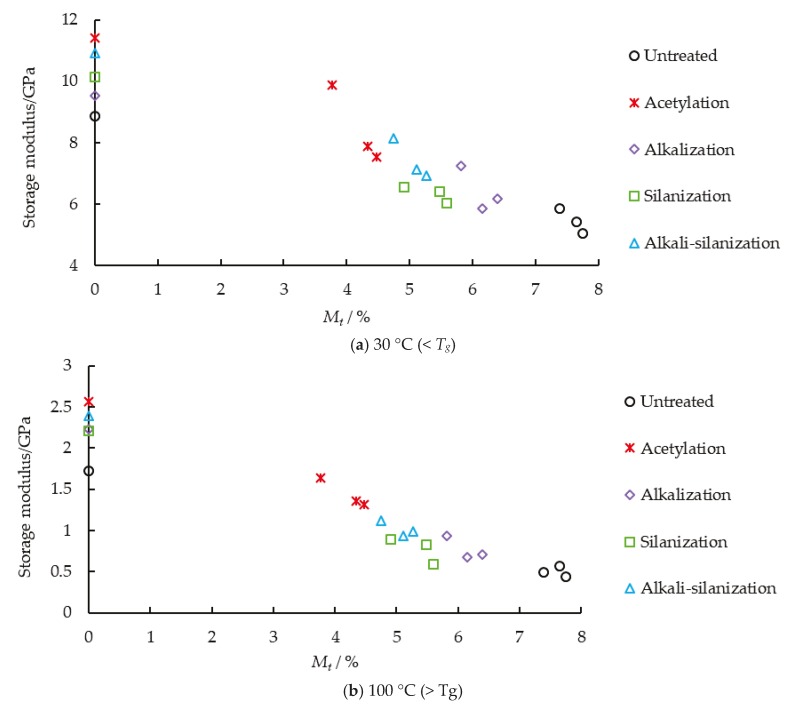
Relationship between storage modulus and moisture content.
